# Single-step genomic prediction of fruit-quality traits using phenotypic records of non-genotyped relatives in citrus

**DOI:** 10.1371/journal.pone.0221880

**Published:** 2019-08-29

**Authors:** Atsushi Imai, Takeshi Kuniga, Terutaka Yoshioka, Keisuke Nonaka, Nobuhito Mitani, Hiroshi Fukamachi, Naofumi Hiehata, Masashi Yamamoto, Takeshi Hayashi

**Affiliations:** 1 Institute of Fruit Tree and Tea Science, National Agriculture and Food Research Organization, Fujimoto, Tsukuba, Ibaraki, Japan; 2 Graduate School of Life and Environmental Science, University of Tsukuba, Tennodai, Tsukuba, Ibaraki, Japan; 3 Western Region Agricultural Research Center, National Agriculture and Food Research Organization, Senyucho, Zentsuji, Kagawa, Japan; 4 Institute of Fruit Tree and Tea Science, National Agriculture and Food Research Organization, Okitsunakacho, Shimizu, Shizuoka, Japan; 5 Nagasaki Agricultural and Forestry Technical Development Center, Nagasaki Prefectural Government, Kaizumachi, Isahaya, Nagasaki, Japan; 6 Faculty of Agriculture, Kagoshima University, Korimoto, Kagoshima, Kagoshima, Japan; 7 Institute of Crop Science, National Agriculture and Food Research Organization, Kannondai, Tsukuba, Ibaraki, Japan; USDA-ARS Southern Regional Research Center, UNITED STATES

## Abstract

The potential of genomic selection (GS) is currently being evaluated for fruit breeding. GS models are usually constructed based on information from both the genotype and phenotype of population. However, information from phenotyped but non-genotyped relatives can also be used to construct GS models, and this additional information can improve their accuracy. In the present study, we evaluated the utility of single-step genomic best linear unbiased prediction (ssGBLUP) in citrus breeding, which is a genomic prediction method that combines the kinship information from genotyped and non-genotyped relatives into a single relationship matrix for a mixed model to apply GS. Fruit weight, sugar content, and acid content of 1,935 citrus individuals, of which 483 had genotype data of 2,354 genome-wide single nucleotide polymorphisms, were evaluated from 2009–2012. The prediction accuracy of ssGBLUP for genotyped individuals was similar to or higher than that of usual genomic best linear unbiased prediction method using only genotyped individuals, especially for sugar content. Therefore, ssGBLUP could yield higher accuracy in genotyped individuals by adding information from non-genotyped relatives. The prediction accuracy of ssGBLUP for non-genotyped individuals was also slightly higher than that of conventional best linear unbiased prediction method using pedigree information. This indicates that ssGBLUP can enhance prediction accuracy of breeding values for non-genotyped individuals using genomic information of genotyped relatives. These results demonstrate the potential of ssGBLUP for fruit breeding, including citrus.

## Introduction

Genomic selection (GS) is considered to be a practical tool for accelerating genetic improvement in plant breeding [1,2], and the potential of GS is now being evaluated for use in fruit breeding [3]. Conventional phenotypic selection in fruit breeding has difficulties owing to long juvenile periods and complex inheritance of quantitative traits [4], and GS is expected to be an alternative method to phenotypic selection and work toward solving these problems.

In plant breeding, statistical GS models are generally constructed based on information from both the genotypes and phenotypes of a population [5]. However, phenotypic data from non-genotyped relatives can also be used to construct GS models when full pedigree records are available [6]. This situation is common in fruit breeding because an organized fruit breeding program has a well-defined recording system and continuously accumulates phenotypic records along with pedigree information, such as in [7,8]. Therefore, phenotypic and pedigree information from non-genotyped relatives could be used to improve the accuracy of GS modeling in fruit breeding.

For GS in animal breeding, phenotypic data from non-genotyped relatives are often incorporated to obtain regular breeding values for genotyped individuals using pedigree information and, subsequently, genomic prediction model is constructed by combining the estimated breeding values and genotypes via multiple steps [9,10]. This procedure is called multiple-step GS, which can be complicated to perform, and can result in lower accuracy, biased outputs, or loss of information [11]. In contrast to multiple-step GS, single-step genomic best linear unbiased prediction (ssGBLUP) has been proposed [11,12], where phenotypic data from both genotyped and non-genotyped individuals are jointly analyzed to predict breeding values of all individuals using a mixed linear model with a relationship matrix obtained by combining genomic relationship information among genotyped individuals and pedigree information between genotyped and non-genotyped individuals and within non-genotyped individuals [13]. Thus, ssGBLUP can predict the breeding values of both genotyped and non-genotyped individuals simultaneously, with lower bias and increased accuracy compared to multiple-step methods [14,15]. Therefore, ssGBLUP could be a promise tool in fruit breeding.

In the procedure of ssGBLUP, a combined relationship matrix, denoted as **H** matrix, is computed from a genome relationship matrix and a pedigree-based relationship matrix, referred to as **G** matrix and **A** matrix, respectively, to fit the best linear unbiased prediction (BLUP) model [13]. Through the **H** matrix, **A** is augmented by **G** and vice versa, enabling ssGBLUP to improve accuracy in the evaluation of breeding values for both genotyped and non-genotyped relatives. However, although ssGBLUP has several advantages to the multiple-step method as described above, the application of this method for plant breeding has been limited to several species, including rice (*Oryza sativa* L.) [16,17], wheat (*Triticum aestivum* L.) [18,19], maize (*Zea mays* L.) [20], and those of forest trees [21–24], and to the best of our knowledge, no previous studies of ssGBLUP have reported for fruit breeding. Accordingly, we applied ssGBLUP to a real dataset of fruit-quality traits obtained from an ongoing citrus breeding program. We compared the prediction accuracy of ssGBLUP with that of conventional methods in both genotyped and non-genotyped individuals.

## Materials and methods

### Plant materials and phenotypic records

**Fig 1:**
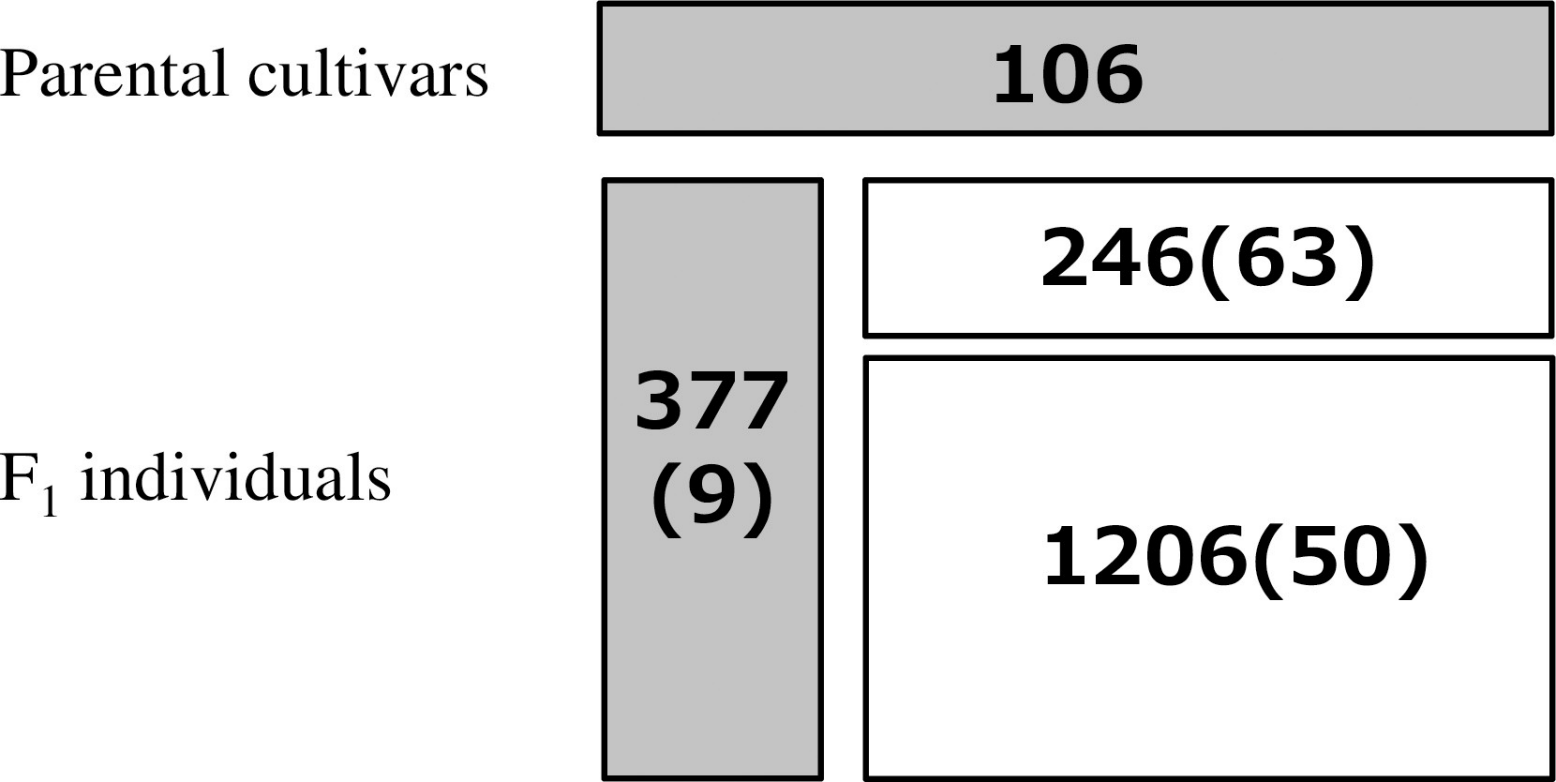
Outline of plant materials used in this study (parental cultivars, 106; F_1_ individuals, 1829; total, 1935). F_1_ individuals were derived from crosses between two parental cultivars. Numbers in the boxes indicate number of individuals in each category described below. Gray and white boxes represent with or without single nucleotide polymorphism (SNP) data, respectively; 483 individuals (106 parental cultivars and 377 F_1_ individuals) have SNP data. Numbers in parentheses represent the number of pair-cross families; thus, e.g., 377 F_1_ individuals with SNP data were derived from nine pair-cross families. F_1_ individuals without SNP data were divided into two categories: those derived from pair-cross families that had less than 10 F_1_ individuals (upper) or more than 10 F_1_ individuals (lower). Family means of the phenotypic records of the latter category were targeted for cross-validation of non-genotyped individuals.

An outline of the plant materials tested is shown in Fig 1. A total of 1935 individuals were obtained from the Kuchinotsu Citrus Research Station, National Agriculture and Food Research Organization (NARO, Nagasaki, Japan). We used 106 parental cultivars and 1829 F_1_ individuals derived from 122 pair-cross families (hereafter, referred to as families). Both the parental cultivars and the F_1_ individuals were maintained as previously described [25]: briefly, the F_1_ individuals were each grafted onto one tree of trifoliate orange (*Poncirus trifoliata* L.) from 2006–2008, which were planted in the breeding fields at a spacing of 0.3 m within and 5 m between rows. Parental cultivars were grafted onto trifoliate orange or satsuma mandarin (*Citrus unshiu* Marcow.) interstocks in adjacent fields. Crosses were performed solely for producing commercial cultivars, and therefore, no specific mating design was adopted. All trees were maintained in accordance with the standard management protocol in Japan, namely, four applications of fertilizer and 10–20 applications of agrichemicals per year.

Three fruit-quality traits including fruit weight (FW), sugar content (SC), and acid content (AC) were evaluated in each tree of the genotypes used in this study (Table 1). Five colored fruits were sampled for immediate evaluations in December, and FW, SC, and AC were determined annually from 2009–2012. Thus, all 1935 individuals were evaluated one–four times for each trait. These phenotypic records were collected through the selection process of our citrus breeding program, NARO, and are summarized in Table 2.

**Table 1:** Phenotypic traits evaluated in this study.

Trait	Abbreviations	Data type	Measurement unit
Fruit weight	FW	Continuous	mean weight of mature fruits (g)
Sugar content	SC	Continuous	mean Brix of juice (Brix%)
Acid content	AC	Continuous	mean citric acid concentration of juice (%)

**Table 2:** Summary statistics of the phenotypic records evaluated in this study.

Year	Descriptive statistics	Traits		
		FW (g)	SC (Brix%)	AC (%)
2009	Mean	141.5	11.8	1.18
	S.D.	62.2	1.5	0.53
	Min	29.7	8.4	0.46
	Max	626.0	16.6	3.37
	Records	390	389	389
2010	Mean	154.7	12.0	1.28
	S.D.	65.5	1.5	0.54
	Min	30.6	8.2	0.54
	Max	621.5	18.3	3.93
	Records	567	565	562
2011	Mean	192.4	10.9	1.20
	S.D.	82.4	1.4	0.52
	Min	38.2	7.4	0.41
	Max	949.5	16.2	3.54
	Records	592	592	592
2012	Mean	155.2	11.6	1.45
	S.D.	60.2	1.3	0.64
	Min	32.8	7.7	0.58
	Max	541.0	16.2	4.48
	Records	1641	1636	1637

*FW* fruit weight, *SC* sugar content, *AC* acid content

### Marker genotypes

All 106 parental cultivars and 377 F_1_ individuals derived from nine families were genotyped using the genotyping-by-sequencing (GBS) method [26] to obtain genome-wide single nucleotide polymorphism (SNP) data. Accordingly, 483 of 1935 individuals have SNP data (outlined in Fig 1). The obtained SNP data were subsequently subjected to quality control (QC) procedures: briefly, the SNP loci were removed with a call rate <0.80 and a minor allele frequency <0.01. The remaining SNPs were further filtered based on the consistency of Mendelian inheritance, and missing SNP genotypes were imputed by Fimpute v. 2.2 [27]. Following the imputation process, highly correlated SNP loci were eliminated according to Wiggans et al. [28]. The detailed GBS conditions and QC procedure, including the extent of linkage disequilibrium (LD) were described previously by Imai et al. [29].

### Prediction models

The following linear mixed model was applied to compare the prediction performance of ssGBLUP with that of genomic best linear unbiased prediction (GBLUP) in genotyped individuals and that of conventional BLUP (ABLUP) in non-genotyped individuals:

y=Xb+Zu+e, (1)

where **y** is a vector of phenotypic records of the 1935 individuals observed from 2009–2012, **b** is a vector of fixed effects including an intercept and year effect, **X** is a design matrix relating **b** to **y**, and **Z** is an incidence matrix relating **u** to **y**. The vector **u** represents breeding values as described below, and **e** is a vector of residuals assuming $e∼N(0,I\sigma_{e}^{2})$, where **I** is an identity matrix and $\sigma_{e}^{2}$ represents residual variance.

In Eq (1), **u** are assumed to follow a normal distribution with a mean vector of 0 and a covariance matrix $A\sigma_{u}^{2}$ in the ABLUP model and $G\sigma_{u}^{2}$ in the GBLUP model, where $\sigma_{u}^{2}$ is the additive genetic variance, and **A** and **G** represent a pedigree-based additive relationship matrix and a realized genomic matrix, respectively. We calculated **G** from SNP data according to VanRaden’s first method [6]. Using the **A** and **G** matrices, the best linear unbiased predictor of **u**, denoted by $\hat{u}$, was calculated for ABLUP as follows:

$$\hat{u}={\left(Ζ'MZ+\lambda A^{-1}\right)}^{-1}Ζ'My,$$ (2)

and for GBLUP as follows:

$$\hat{u}={\left(Ζ'MZ+\lambda G^{-1}\right)}^{-1}Ζ'My,$$ (3)

where *λ* is given as $\lambda =\sigma_{e}^{2}/\sigma_{u}^{2}$, and **M** is a projection matrix defined as **M** = **I**−**X**(**X**'**X**)^−1^**X**'. The **A** and **G** matrices were computed using airemlf90 [30] and preGSf90 software [12,31], respectively, and $\hat{u}$ was calculated using airemlf90 software [30].

In the ssGBLUP model, it was assumed that $u∼N(0,H\sigma_{u}^{2})$ in Eq (1). This **H** matrix combines pedigree and genomic relationships, and was defined previously [11] as follows:

$$H=\left[\begin{array}{cc} A_{11}+A_{12}A_{22}^{-1}(G-A_{22})A_{22}^{-1}A_{21} & A_{12}A_{22}^{-1}G\\ GA_{22}^{-1}A_{21} & G \end{array}\right],$$ (4)

where **A**_11_, **A**_12_, **A**_21_, and **A**_22_ are submatrices of **A**, and the subscripted 1 and 2 represent non-genotyped and genotyped individuals, respectively. Through the **H** matrix, the prediction accuracy of genotyped individuals can be improved with data from non-genotyped relatives, and the prediction accuracy of non-genotyped individuals can also be improved by **G**, which accounts for the Mendelian sampling effect of genotyped relatives and can provide more accurate relationships than **A**. For the **H** matrix calculation, we scaled **G** based on **A**_22_ so that the mean diagonal and off-diagonal of **G** equals those of **A**_22_; appropriate scaling avoids the biases of breeding values in genotyped individuals [14]. The inverse of **H** has a simple form [12,32], and can be written with tuning-parameters α, β, τ, and ω as follows:

$$H^{-1}=A^{-1}+\left[\begin{array}{cc} 0 & 0\\ 0 & \tau {\left(\alpha G+\beta A_{22}\right)}^{-1}-\omega A_{22}^{-1} \end{array}\right].$$ (5)

Fine tuning of α, β, τ, and ω can increase the accuracy and reduce biases of genomic prediction of breeding values [33]. We used fixed values of α = 0.95 and β = 0.05 to enable inversion of the matrix. We assigned the same value to τ and ω (τ = ω); in this context, τ defines a mixing proportion of genomic and pedigree information [12]. If τ > 0, and τ = ω, then the portion of genomic and pedigree information becomes *τ*:(1−*τ*). Adding pedigree information could be beneficial for capturing the polygenic effects that could not to be accounted for by genomic information. We tested three values of τ (1.00, 0.75, and 0.50) for evaluation of prediction accuracy. Using the **H** matrix, the best linear unbiased predictor $\hat{u}$ was calculated as follows:

$$\hat{u}={\left(Ζ'MZ+\lambda H^{-1}\right)}^{-1}Ζ'My,$$ (6)

where *λ* and **M** are defined the same as the ABLUP and GBLUP models. The **H** matrices were computed using the preGSf90 software [12,31], and $\hat{u}$ was calculated using airemlf90 software [30].

### Heritability estimation

Additive genetic variance ($\sigma_{u}^{2}$), residual variance ($\sigma_{e}^{2}$), and heritability ${(h}^{2}=\sigma_{u}^{2}/(\sigma_{u}^{2}+\sigma_{e}^{2}))$ in each trait were estimated based on the linear mixed model described above. We estimated the heritability by ABLUP and ssGBLUP with different τ values (1.00, 0.75, and 0.50). We did not calculate the heritability by the GBLUP method, because GBLUP only applied to the dataset with both phenotyped and genotyped individuals.

### Evaluation of prediction accuracy

The prediction accuracy of ssGBLUP was compared with that of GBLUP in genotyped individuals. Cross-validation (CV) was performed to evaluate these methods, assuming early selection at the juvenile stage. CV was also performed to compare the prediction accuracy of ssGBLUP with that of ABLUP in non-genotyped individuals.

To compare the prediction accuracy in genotyped individuals, genotypic values (i.e., sum of the intercept and breeding values) of individuals from nine genotyped families were calculated based on all phenotypic records of the 1935 individuals by the ABLUP method, and these values were predicted by the ssGBLUP and GBLUP methods. In each CV cycle, each of the nine genotyped families was omitted and the remaining individuals, including the parental cultivars and non-genotyped families (only in ssGBLUP), were used to construct the prediction model to predict the genotypic values of the omitted family. Thus, CV consisted of nine cycles and evaluated the accuracy of seedling selection based on SNP genotypes at the juvenile stage during cross-breeding. The prediction accuracy was evaluated as a correlation coefficient (*r*) between the targeted genotypic values and the predicted ones.

To compare the prediction accuracy in non-genotyped individuals, phenotypic mean values in each of the 50 non-genotyped families with more than 10 F_1_ individuals (hereafter, referred to as observed family mean) were calculated as the target values of the CV procedures. These values were predicted by ssGBLUP and ABLUP methods, which calculated the predicted genotypic values in each target family for validation. The phenotypic records for calculation of the family mean were adjusted for year effect that was estimated from all observations of the 1935 individuals by the ABLUP method. In the ssGBLUP analysis, we adopted the fixed values of τ (ω) with the highest prediction accuracy in genotyped individuals. In each CV cycle, each of the 50 non-genotyped families were omitted and the remaining individuals, including the parental cultivars, genotyped families, and non-genotyped families with less than 10 F_1_ individuals, were used to construct the prediction model to predict the observed means of the omitted family. In this case, the predicted genotypic values became identical within a family, because their phenotypic records were omitted. The prediction accuracy was evaluated as weighted correlation coefficient (*r*) between the target and predicted values. The weights of the correlation coefficient were determined from the numbers of F_1_ individuals in each family.

## Results

### Heritability estimation

We estimated heritability using ABLUP and ssGBLUP with three τ values (Table 3). Heritability ranged from 0.57 to 0.82 in three fruit-quality traits, and AC showed the highest estimates of heritability. These estimates were somewhat lower than those from our previous report [25], which reflects the differences in population to be analyzed. In all traits, ABLUP and ssGBLUP offered almost the same heritability estimates. The mixing proportion τ of ssGBLUP also had little effect on heritability estimation, thus we considered GBLUP provided similar estimates of heritability in our case.

**Table 3:** Heritability estimated by ABLUP and ssGBLUP methods.

Method	τ (ω)^a^	Heritability		
		FW	SC	AC
ABLUP	–	0.61	0.57	0.81
ssGBLUP	1.00	0.63	0.58	0.82
	0.75	0.63	0.58	0.82
	0.50	0.62	0.58	0.81

*FW* fruit weight, *SC* sugar content, *AC* acid content

*ABLUP* best linear unbiased prediction with pedigree-based additive relationships, *ssGBLUP* single-step genomic BLUP

^a^ mixing proportion of genomic information with pedigree information

### Comparison of prediction accuracy in genotyped individuals

The GBS approach and successive QC procedures provided 2353 SNPs from 483 individuals. Using the SNP data and pedigree information of all individuals, we constructed **H** matrices and applied them to the ssGBLUP to evaluate the prediction accuracy in genotyped individuals and to compare with those of GBLUP. For **H** matrix construction, we used the three values of τ (1.00, 0.75, and 0.50), which define the mixing proportion of genomic and pedigree information. Thus, we compared three ssGBLUP models with different τ values and one GBLUP model for three fruit-quality traits.

The CV for each genotyped family showed a similar or higher accuracy in ssGBLUP compared with GBLUP (Table 4; S1–S3 Figs). While our result showed rather lower prediction accuracy for GBLUP than that of previous study that evaluated the same traits [34], the reduced accuracy may be caused by the differences in SNPs, plant materials, and the procedures of CV. A considerable improvement in accuracy was attained in SC, and similar accuracy was obtained in FW and AC. The comparisons between the ssGBLUP models with different τ values showed that the highest accuracy was obtained when τ = 1.00 for FW, 0.50 for SC, and 0.75 for AC (Table 4). However, the differences in accuracy were small and showed little effect on the accuracy of ssGBLUP.

**Table 4:** Comparison of prediction accuracy between ssGBLUP and GBLUP methods in genotyped individuals.

Method	τ (ω)^a^	FW	SC	AC
GBLUP	–	0.642 (0.040)^b^	0.432 (0.047)	0.655 (0.039)
ssGBLUP	1.00	**0.650** (0.039)	0.512 (0.044)	0.661 (0.039)
	0.75	0.648 (0.039)	0.516 (0.044)	**0.666** (0.039)
	0.50	0.642 (0.040)	**0.519** (0.044)	0.654 (0.039)

*FW* fruit weight, *SC* sugar content, *AC* acid content

^a^ mixing proportion of genomic information with pedigree information

^b^ Pearson’s correlation coefficients measured as the prediction accuracy in genotyped individuals. Highest coefficients are shown in bold. Numbers in parentheses are standard errors.

### Comparison of prediction accuracy in non-genotyped individuals

The **H** matrix in ssGBLUP combined pedigree and genomic relationships. Consequently, this method could provide more accurate genetic evaluation even for non-genotyped relatives than ABLUP method only using a pedigree-based additive relationship matrix. We validated the prediction accuracy of the ssGBLUP and ABLUP methods. The observed family means in each of the 50 non-genotyped families were predicted using CV procedures for each trait. Slightly higher correlation coefficients resulted from the ssGBLUP method compared to those of the ABLUP method (Table 5; S4–S6 Figs). The improvement in prediction accuracy achieved by ssGBLUP was higher for SC than it was for FW and AC. Although the prediction accuracy was considerably different for each trait, large discrepancies between the observed and predicted values were commonly detected in several families.

**Table 5:** Comparison of prediction accuracy between ABLUP and ssGBLUP method in non-genotyped individuals.

Method	FW	SC	AC
ABLUP	0.294 (0.134)^b^	0.498 (0.125)	0.771 (0.091)
ssGBLUP^a^	**0.295** (0.138)	**0.538** (0.121)	**0.783** (0.090)

*FW* fruit weight, *SC* sugar content, *AC* acid content

^a^ The mixing proportion (τ) that showed the highest prediction accuracy in comparison with GBLUP method were used (1.00 for FW, 0.50 for SC, and 0.75 for AC, respectively. See Table 4).

^b^ Weighted Pearson’s correlation coefficients measured as the prediction accuracy in non-genotyped individuals. Weights are determined by number of progeny in each combination.

Highest coefficients are shown in bold. Numbers in parentheses are standard errors.

## Discussion

Recently, GS has attracted the attention of those involved in fruit breeding, because it has the potential to capture minor gene effects, and thus provide more accurate selection of complex quantitative traits of economic importance [34–37]. However, to construct reliable models for GS, a sufficiently large training population with both genotyped and phenotyped individuals is required [38,39]. This is one of the main obstacles for the introduction of GS for fruit breeding, because a long juvenile period and large plant size hinders the rapid accumulation of phenotypic data such as fruit-quality traits. In addition, the genotypic data necessary for GS can only be obtained from living individuals, although most individuals evaluated in breeding programs are culled after selection. Thus, obtaining both genotype and phenotype records for GS model construction is more difficult for fruit breeding than it is for animal breeding or other crop breeding.

One possible solution for constructing reliable GS models in fruit breeding would be to use previously accumulated phenotype records, e.g., from the breeding procedure, which can be achieved using the ssGBLUP methodology. Generally, an organized fruit breeding program includes well-defined maintenance protocols for the breeding materials [40], and phenotyping protocols [41–43]. These practices enable the continuous accumulation of phenotypes and other records that are useful for breeding such as those containing pedigree information [7,8,25]. Therefore, ssGBLUP can be introduced into fruit breeding programs with few changes to the existing system for maintenance of breeding materials and phenotypic evaluations.

In the present study, we compared the prediction performance of ssGBLUP with that of GBLUP, assuming selection at the juvenile stage in the genotyped individuals. We also compared the prediction performance of ssGBLUP with that of conventional ABLUP in the non-genotyped individuals. Our results showed that ssGBLUP equaled or outperformed GBLUP and ABLUP in terms of prediction accuracy in all cases, especially for SC. These gains in prediction accuracy were consistent with those from previous reports on different plants, such as rice [16,17] and wheat [18,19], and on domesticated animals including dairy cattle [44,45], beef cattle [46,47], pigs [15], and chickens [48]. With the **H** matrix, genomic information that can account for Mendelian sampling is incorporated into standard BLUP models. Furthermore, much larger datasets of phenotypic information can be used with the ssGBLUP method than with the GBLUP method. These advantages of using the ssGBLUP method are herein confirmed for citrus.

Although we have demonstrated the potential of ssGBLUP for use in citrus breeding, there remains several problems. For CV of non-genotyped individuals, large discrepancies between observed and predicted family means were detected (S4–S6 Figs). These large discrepancies indicate that predictions from the ssGBLUP method could be inaccurate in some cases, at least for fruit-quality traits in citrus. One possible cause of these large discrepancies may be the influence of non-additive effects, such as dominance or epistasis effects. Under the assumption of an infinitesimal model [49], ssGBLUP assumes additive polygenic effects as the mode of inheritance for target traits. Although the assumption of additive effect captures a large part of dominant and epistasis effects [50,51], the predictions from ssGBLUP may, in some cases, have some outliers that are affected by large non-additive effects, despite moderate to high narrow-sense heritability traits as analyzed in our study.

In addition to the problems from non-additive effects, several previous studies have reported factors that influence the accuracy of genomic predictions, including training population size, heritability, genetic architecture of target traits, extent of LD, and marker density [39,52–54]. For these factors, the extent of LD determines the marker density necessary for genomic predictions, and an insufficient number of SNP markers against LD decreases the model’s prediction accuracy due to imperfect associations between quantitative trait loci (QTL) and SNP markers. Our previous study and others have reported relatively high LD in fruit breeding populations [29,34,37]. Thus, a smaller number of SNPs may be sufficient for GS in an advanced fruit breeding population. In addition, for the GBLUP method (and also for ssGBLUP), the effect of increasing the number of SNPs on prediction accuracy can appear to reduce the sampling error of **G**, and a larger number of SNPs would provide only small improvements in accuracy if the effects of QTLs are well captured by a small number of SNPs [33]. However, if it is not the case, it may be desirable to capture polygenic effects using an **A** matrix and tuning the mixing proportion of the **A** and **G** matrices [33]. Nevertheless, our study demonstrated that the τ parameters had little effect on prediction accuracy for the three fruit-quality traits tested. This is in contrast to the results of the first report on ssGBLUP in plants [18], which stated the importance of trait-specific weighting parameters (τ parameters in the present study). Owing to the inconsistent results for τ parameters observed in the previous report and the present study, the effect of τ parameters on prediction accuracy should be carefully considered when they are applied to other traits or other species of fruit.

The accuracy of genomic predictions is also affected by the heritability of the target traits [39]; the higher prediction accuracy is obtained for a trait with higher heritability. For three fruit-quality traits evaluated in this study, AC showed the highest heritability, and showed slightly or considerably higher prediction accuracy compared with the other two traits in both genotyped and non-genotyped individuals (Tables 4 and 5). These results suggested that heritability can be a measure for evaluating prediction accuracy in genomic predictions with ssGBLUP for fruit breeding, although an inconsistent result was observed between FW and SC in non-genotyped individuals. Furthermore, the heritability of target traits is used to estimate the training population size necessary to achieve predetermined accuracy of genomic predictions [55], and a larger population size is necessary if heritability is low. Although ssGBLUP could achieve larger sample sizes compared with those of GBLUP, the greater number of individuals is more desirable for the construction and validation of GS models, especially for low heritability traits. Our study included only moderate to high heritability traits (0.57 to 0.82); therefore, the prediction accuracy of ssGBLUP for lower-heritability traits should be further evaluated with larger datasets in future studies.

As for the genetic architecture of target traits, ssGBLUP assumes additive polygenic inheritance of target traits which are contributed by a large number of QTLs each with small effect. However, several studies have reported the QTLs with large effects in three fruit-quality traits evaluated in our study [29,34,56–58]. Therefore, these genetic architectures may decrease the prediction accuracy of ssGBLUP. As a modified ssGBLUP method, a single-step methodology using Bayesian regression, which can assume different marker variances, was recently proposed by Fernando et al. [59]. Their method can treat large QTL effects which are estimated as marker effects in the prediction model, and thus has the potential to further improve genomic prediction accuracy. Although the studies of Hayes et al. [9] and VanRaden et al. [10] indicated that a suitable number of markers with equal variance is appropriate for most traits, the application of Fernando et al.’s single-step methodology using Bayesian regression may be an alternative choice for GS in fruit breeding.

Although there are still problems to overcome, we have demonstrated the potential of ssGBLUP for fruit breeding using actual data of citrus. We consider that the several features of ssGBLUP methodology, which uses information from both genotyped and non-genotyped relatives with simple manners, makes it suitable for ongoing fruit breeding programs. The advantages of ssGBLUP and other single-step GS approaches can increase in the future with the accumulation of larger phenotypic and genotypic datasets

## Supporting information

S1 Fig Plots of the estimated genotypic values vs. predicted genotypic values via cross-validation in fruit weight. Estimated genotypic values were calculated using a numerator relationship matrix (**A**) including all observations from 1935 individuals. Predicted genotypic values via cross-validation were calculated using a genomic relationships matrix (**G**, GBLUP) or combined **H** matrix from **G** and **A** (single-step GBLUP) excluding phenotypic records of each target family for cross-validation. (a) GBLUP model (b) ssGBLUP model with τ = 0.50 (c) ssGBLUP model with τ = 0.75 (d) ssGBLUP model with τ = 1.00.
(PDF)

S2 Fig Plots of the estimated genotypic values vs. predicted genotypic values via cross-validation in sugar content. Estimated genotypic values were calculated using a numerator relationship matrix (**A**) including all observations from 1935 individuals. Predicted genotypic values via cross-validation were calculated using a genomic relationships matrix (**G**, GBLUP) or combined **H** matrix from **G** and **A** (single-step GBLUP) excluding phenotypic records of each target family for cross-validation. (a) GBLUP model (b) ssGBLUP model with τ = 0.50 (c) ssGBLUP model with τ = 0.75 (d) ssGBLUP model with τ = 1.00.
(PDF)

S3 Fig Plots of the estimated genotypic values vs. predicted genotypic values via cross-validation in acid content. Estimated genotypic values were calculated using a numerator relationship matrix (**A**) including all observations from 1935 individuals. Predicted genotypic values via cross-validation were calculated using a genomic relationships matrix (**G**, GBLUP) or combined **H** matrix from **G** and **A** (single-step GBLUP) excluding phenotypic records of each target family for cross-validation. (a) GBLUP model (b) ssGBLUP model with τ = 0.50 (c) ssGBLUP model with τ = 0.75 (d) ssGBLUP model with τ = 1.00.
(PDF)

S4 Fig Plots of the observed family means vs. predicted family means via cross-validation in fruit weight. Observed family means refer to mean values of phenotypic records, and predicted family means refer to predicted genotypic values in each pair-cross family. Phenotypic records for calculation of observed family means were adjusted for year effects. Predicted values via cross-validation were calculated using a pedigree-based BLUP model (ABLUP) or single-step GBLUP model (ssGBLUP) excluding the phenotypic records of each target family; thus, they offered the same values within a family. Mixing proportion τ showing the highest accuracy in prediction of genotypic values was used for ssGBLUP model. (a) ABLUP model (b) ssGBLUP model with τ = 1.00.
(PDF)

S5 Fig Plots of the observed family means vs. predicted family means via cross-validation in fruit weight. Observed family means refer to mean values of phenotypic records, and predicted family means refer to predicted genotypic values in each pair-cross family. Phenotypic records for calculation of observed family means were adjusted for year effects. Predicted values via cross-validation were calculated using a pedigree-based BLUP model (ABLUP) or single-step GBLUP model (ssGBLUP) excluding the phenotypic records of each target family; thus, they offered the same values within a family. Mixing proportion τ showing the highest accuracy in prediction of genotypic values was used for ssGBLUP model. (a) ABLUP model (b) ssGBLUP model with τ = 0.50.
(PDF)

S6 Fig Plots of the observed family means vs. predicted family means via cross-validation in fruit weight. Observed family means refer to mean values of phenotypic records, and predicted family means refer to predicted genotypic values in each pair-cross family. Phenotypic records for calculation of observed family means were adjusted for year effects. Predicted values via cross-validation were calculated using a pedigree-based BLUP model (ABLUP) or single-step GBLUP model (ssGBLUP) excluding the phenotypic records of each target family; thus, they offered the same values within a family. Mixing proportion τ showing the highest accuracy in prediction of genotypic values was used for ssGBLUP model. (a) ABLUP model (b) ssGBLUP model with τ = 0.75.
(PDF)
