# Supplementary figures and images for: Single-step genomic prediction of fruit-quality traits using phenotypic records of non-genotyped relatives in citrus
Source: PLoS One. 2019 Aug 29;14(8):e0221880. doi: 10.1371/journal.pone.0221880 (PMC6715226; doi:10.1371/journal.pone.0221880)

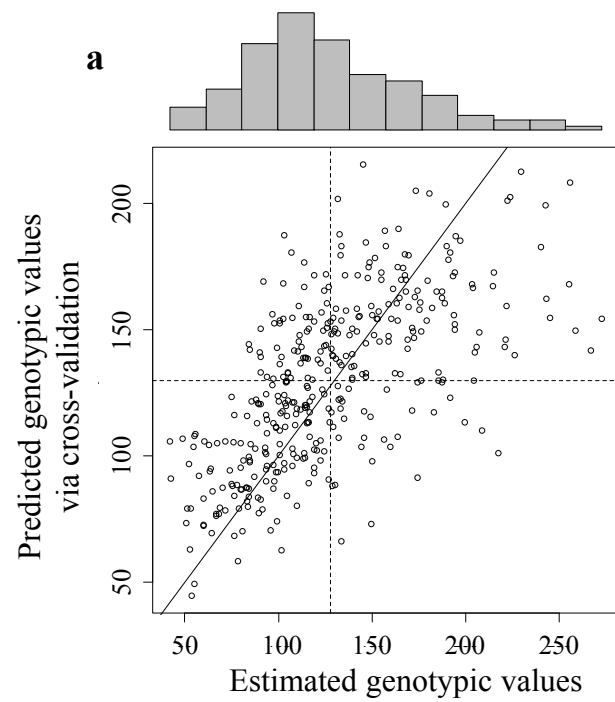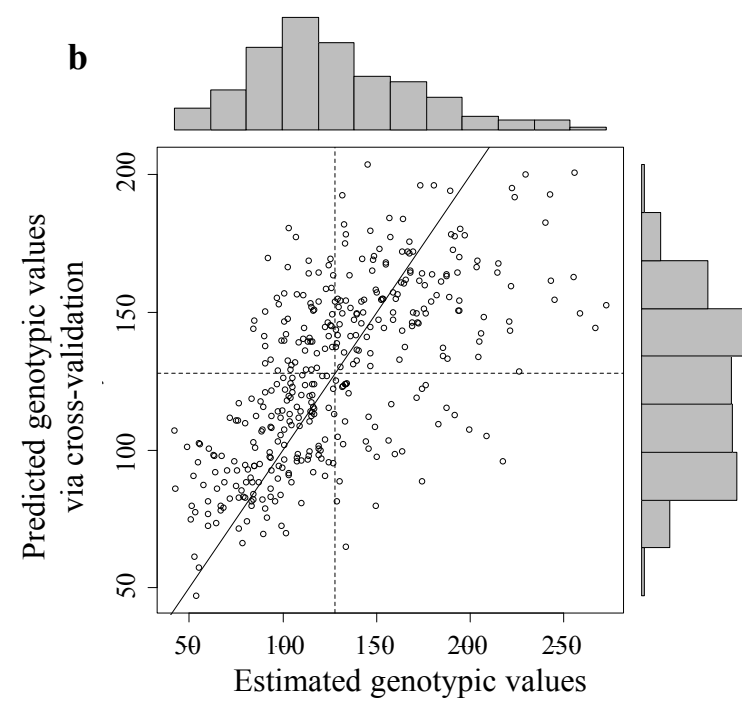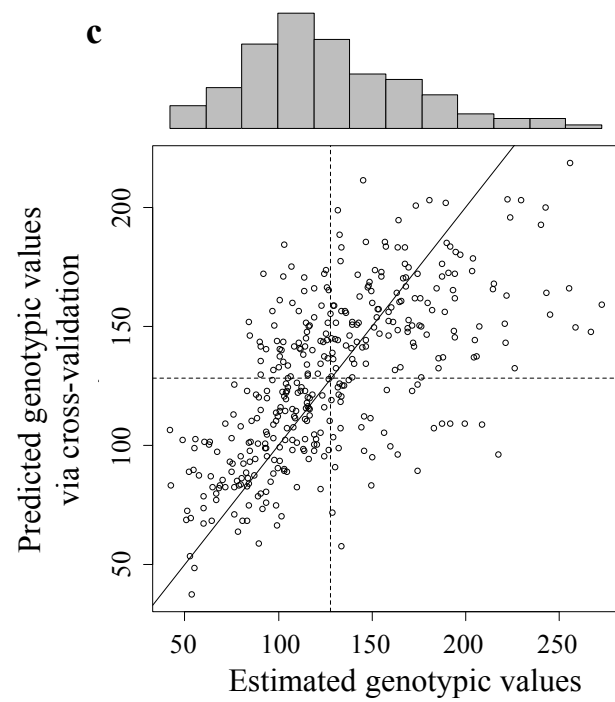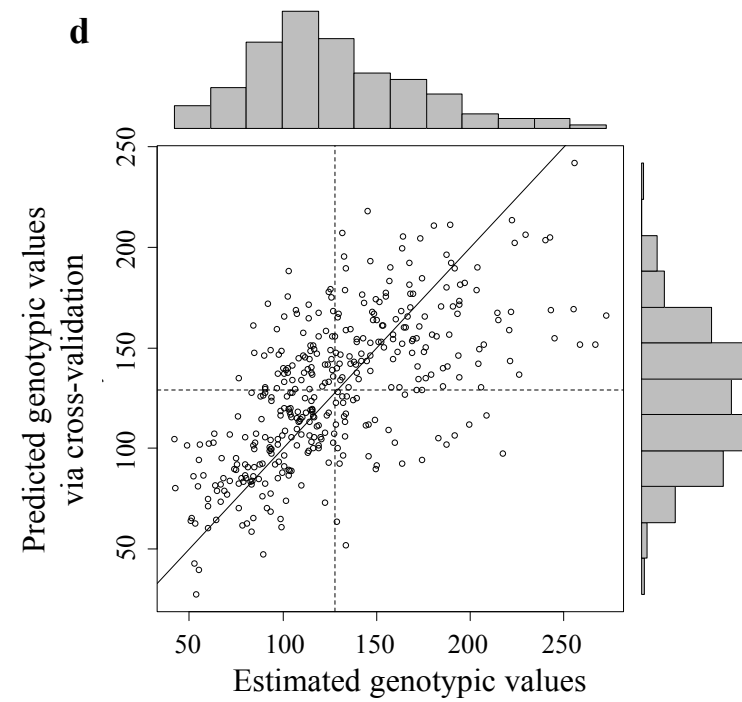

Supplement: S1 Fig — Estimated genotypic values were calculated using a numerator relationship matrix (A) including all observations from 1935 individuals. Predicted genotypic values via cross-validation were calculated using a genomic relationships matrix (G, GBLUP) or combined H matrix from G and A (single-step GBLUP) excluding phenotypic records of each target family for cross-validation. (a) GBLUP model (b) ssGBLUP model with τ = 0.50 (c) ssGBLUP model with τ = 0.75 (d) ssGBLUP model with τ = 1.00. (PDF) [file pone.0221880.s001.pdf]

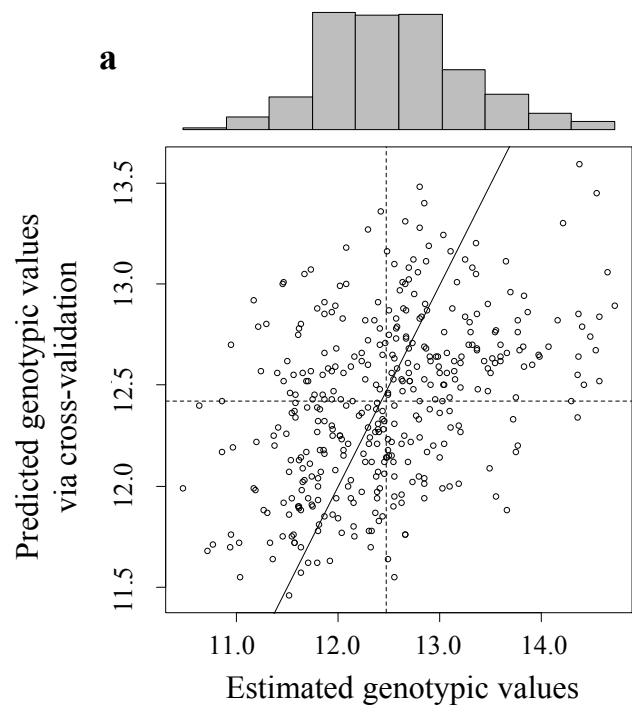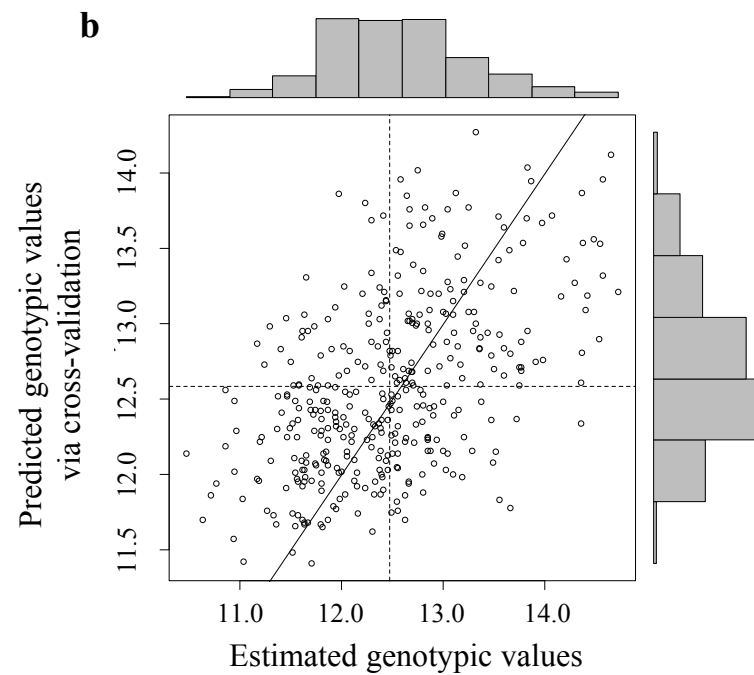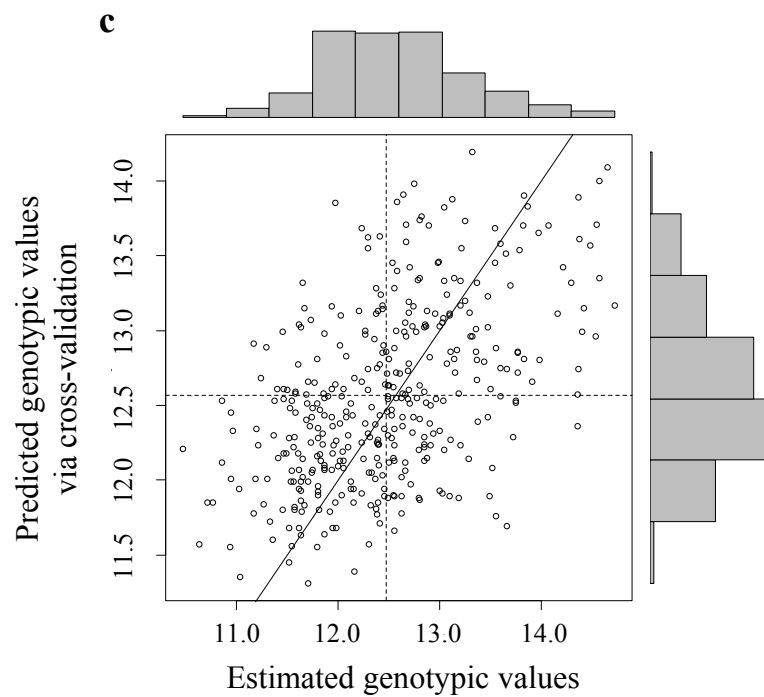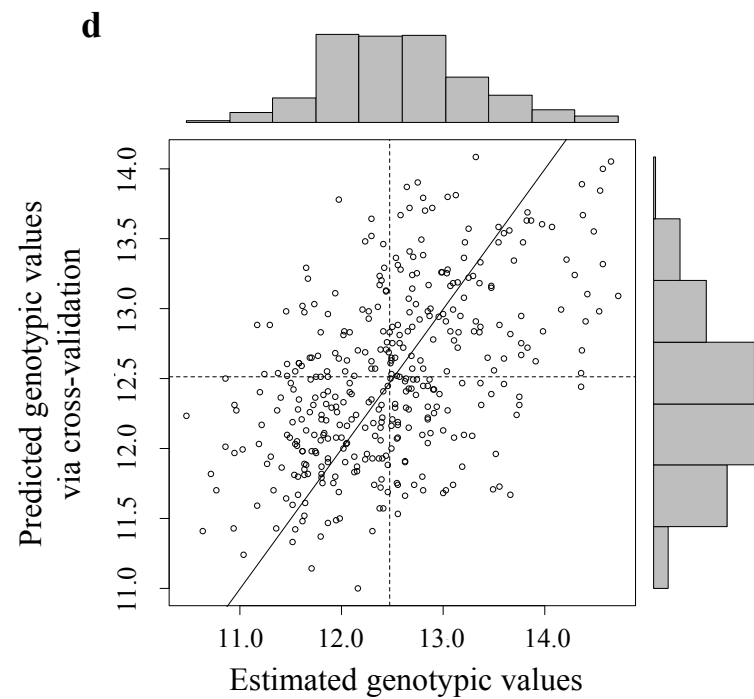

Supplement: S2 Fig — Estimated genotypic values were calculated using a numerator relationship matrix (A) including all observations from 1935 individuals. Predicted genotypic values via cross-validation were calculated using a genomic relationships matrix (G, GBLUP) or combined H matrix from G and A (single-step GBLUP) excluding phenotypic records of each target family for cross-validation. (a) GBLUP model (b) ssGBLUP model with τ = 0.50 (c) ssGBLUP model with τ = 0.75 (d) ssGBLUP model with τ = 1.00. (PDF) [file pone.0221880.s002.pdf]

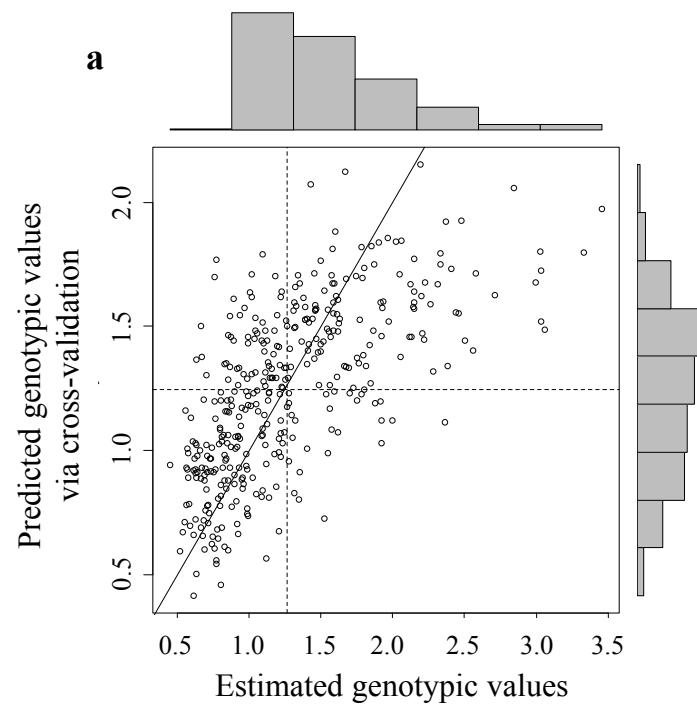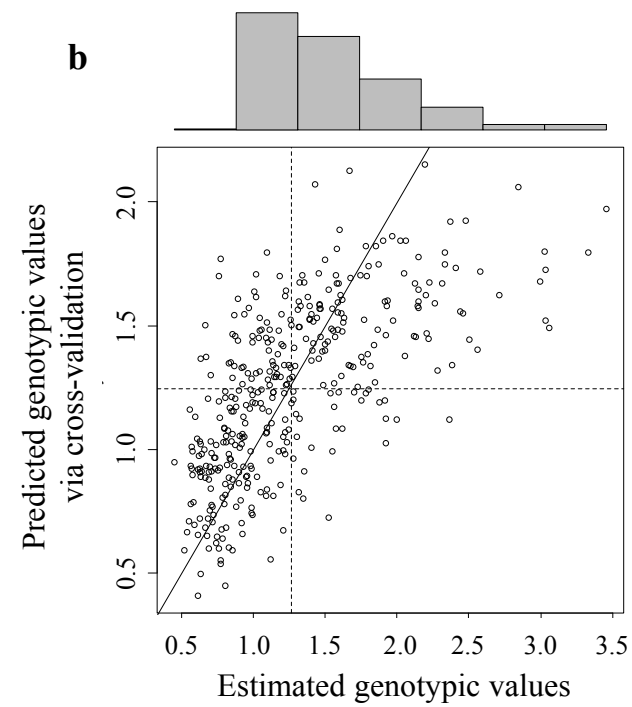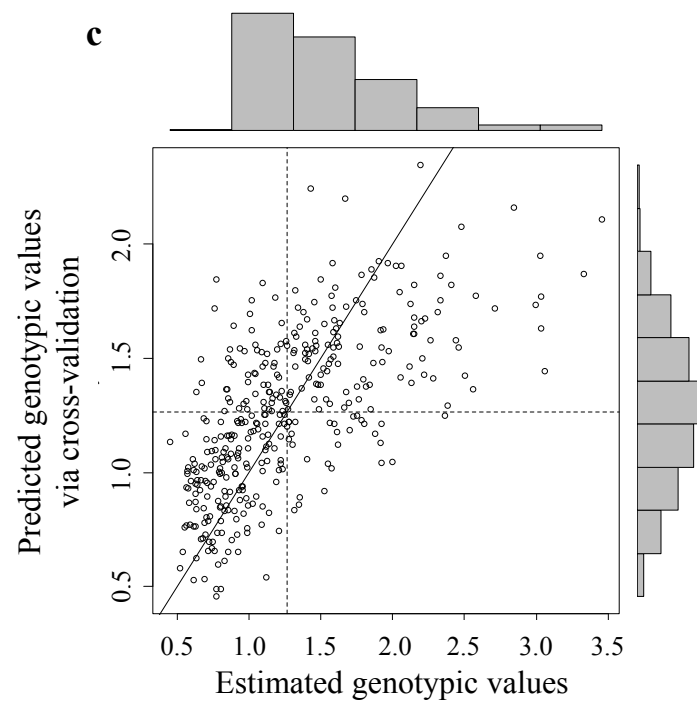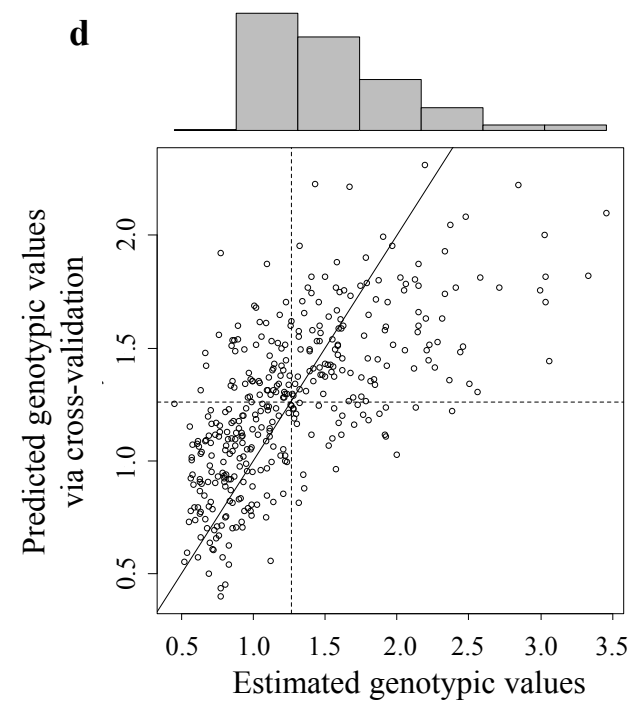

Supplement: S3 Fig — Estimated genotypic values were calculated using a numerator relationship matrix (A) including all observations from 1935 individuals. Predicted genotypic values via cross-validation were calculated using a genomic relationships matrix (G, GBLUP) or combined H matrix from G and A (single-step GBLUP) excluding phenotypic records of each target family for cross-validation. (a) GBLUP model (b) ssGBLUP model with τ = 0.50 (c) ssGBLUP model with τ = 0.75 (d) ssGBLUP model with τ = 1.00. (PDF) [file pone.0221880.s003.pdf]

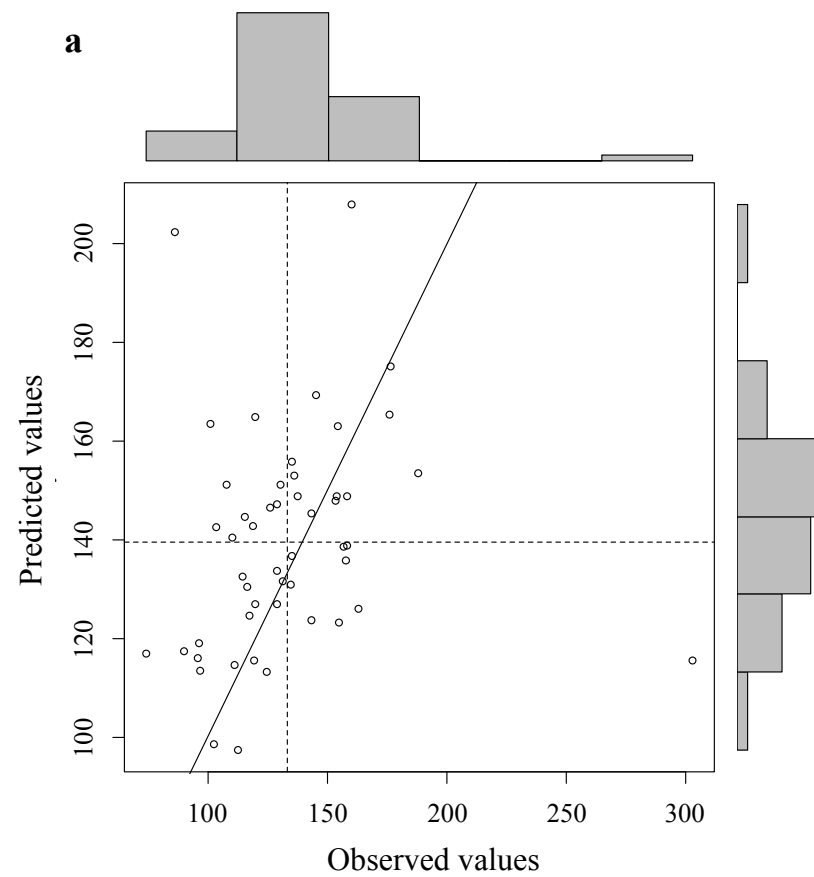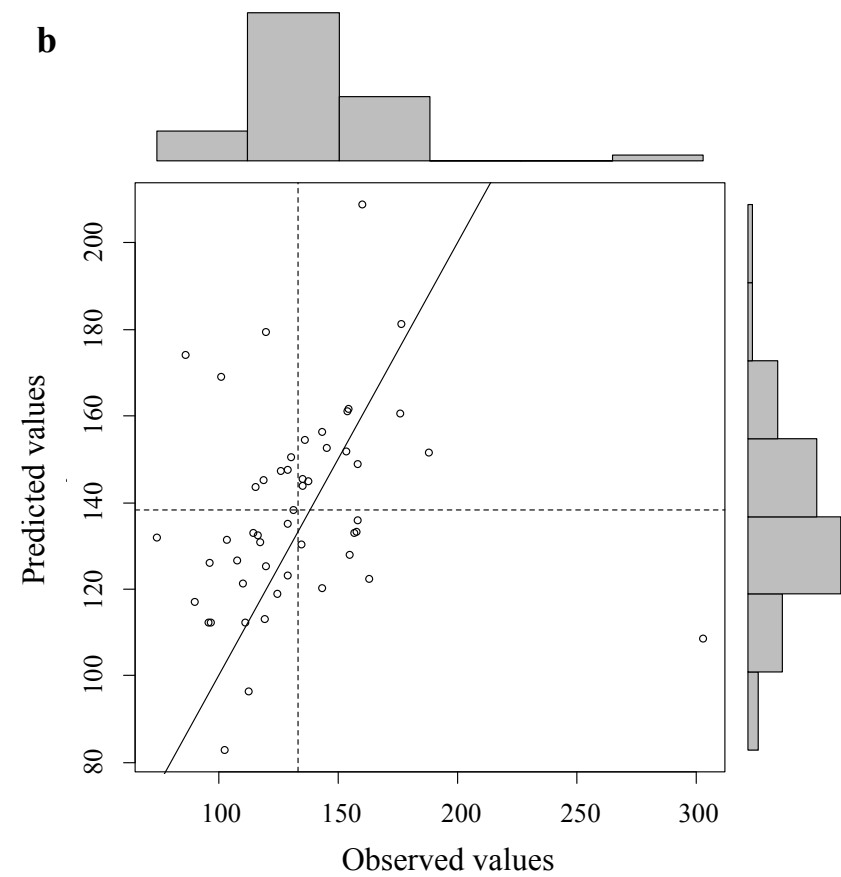

Supplement: S4 Fig — Observed family means refer to mean values of phenotypic records, and predicted family means refer to predicted genotypic values in each pair-cross family. Phenotypic records for calculation of observed family means were adjusted for year effects. Predicted values via cross-validation were calculated using a pedigree-based BLUP model (ABLUP) or single-step GBLUP model (ssGBLUP) excluding the phenotypic records of each target family; thus, they offered the same values within a family. Mixing proportion τ showing the highest accuracy in prediction of genotypic values was used for ssGBLUP model. (a) ABLUP model (b) ssGBLUP model with τ = 1.00. (PDF) [file pone.0221880.s004.pdf]

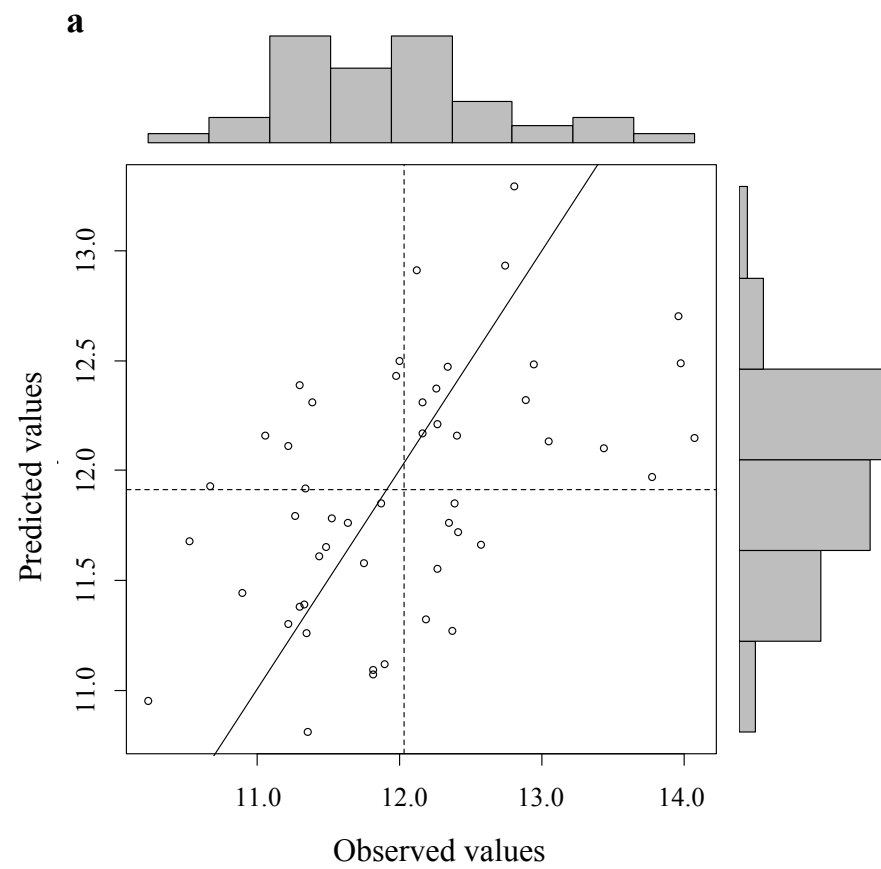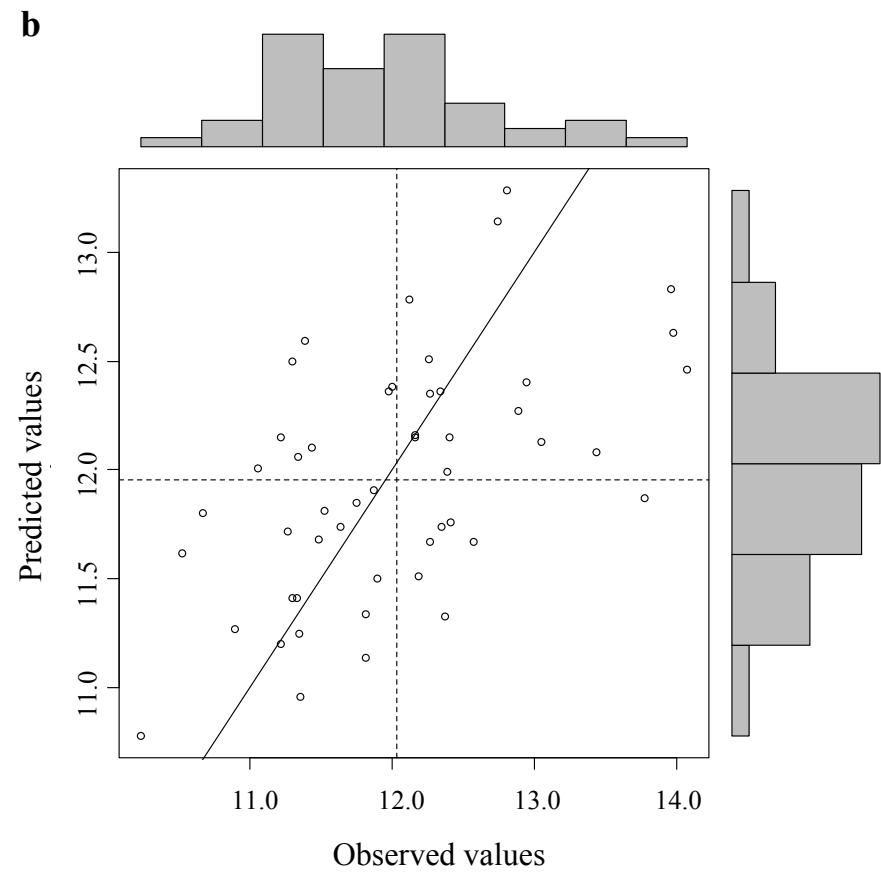

Supplement: S5 Fig — Observed family means refer to mean values of phenotypic records, and predicted family means refer to predicted genotypic values in each pair-cross family. Phenotypic records for calculation of observed family means were adjusted for year effects. Predicted values via cross-validation were calculated using a pedigree-based BLUP model (ABLUP) or single-step GBLUP model (ssGBLUP) excluding the phenotypic records of each target family; thus, they offered the same values within a family. Mixing proportion τ showing the highest accuracy in prediction of genotypic values was used for ssGBLUP model. (a) ABLUP model (b) ssGBLUP model with τ = 0.50. (PDF) [file pone.0221880.s005.pdf]

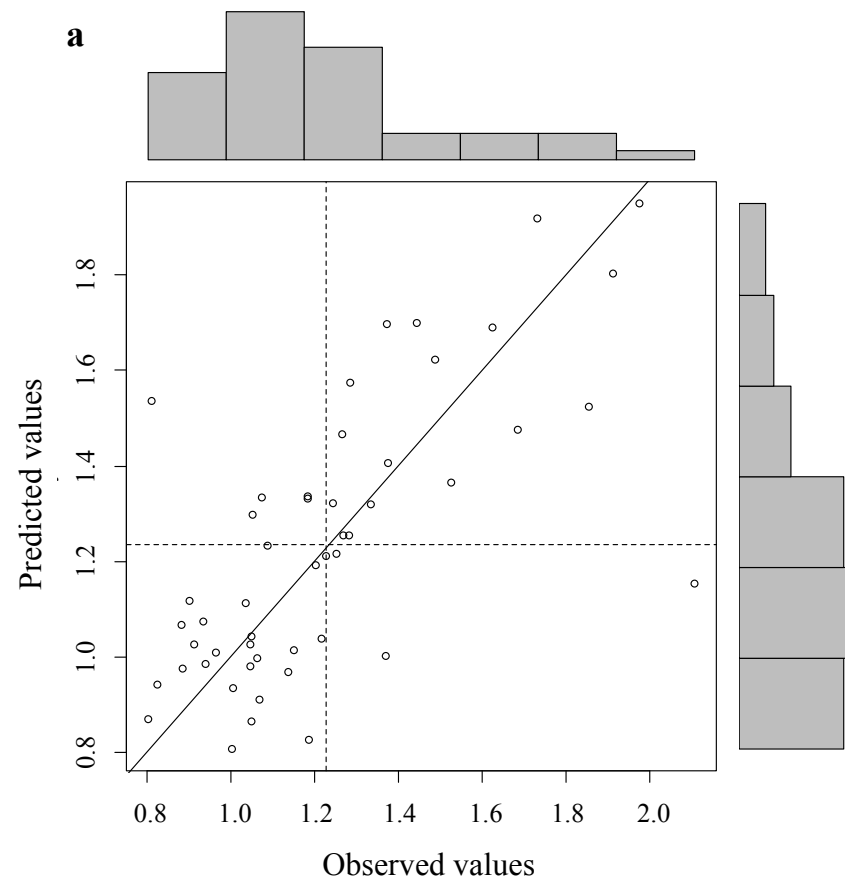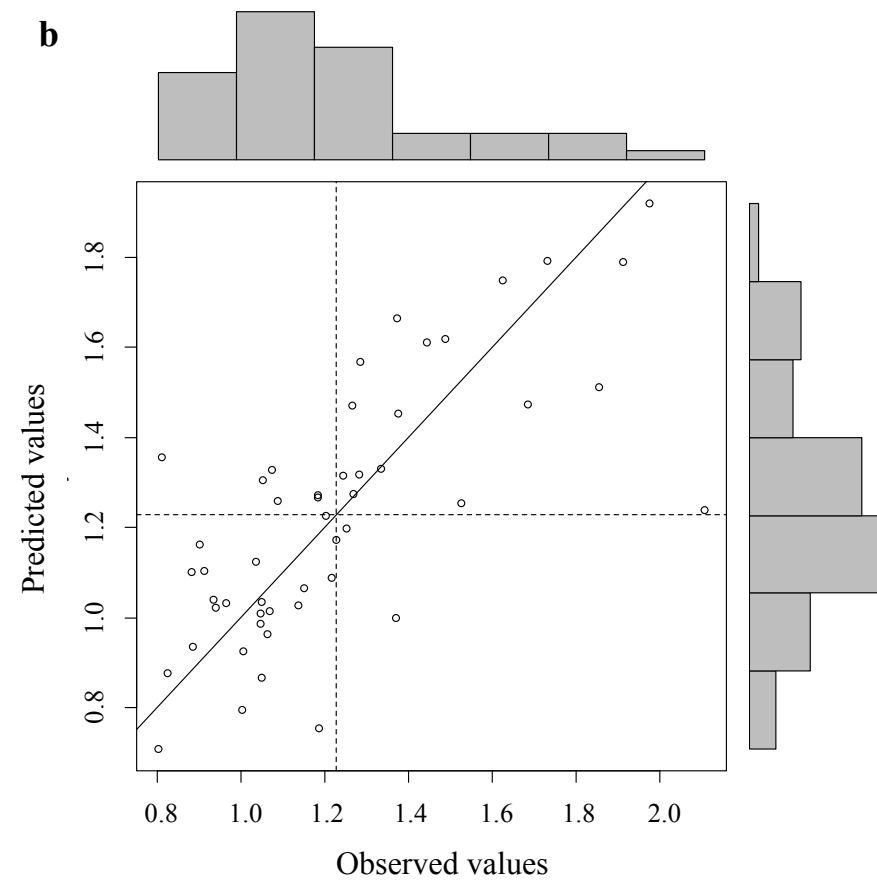

Supplement: S6 Fig — Observed family means refer to mean values of phenotypic records, and predicted family means refer to predicted genotypic values in each pair-cross family. Phenotypic records for calculation of observed family means were adjusted for year effects. Predicted values via cross-validation were calculated using a pedigree-based BLUP model (ABLUP) or single-step GBLUP model (ssGBLUP) excluding the phenotypic records of each target family; thus, they offered the same values within a family. Mixing proportion τ showing the highest accuracy in prediction of genotypic values was used for ssGBLUP model. (a) ABLUP model (b) ssGBLUP model with τ = 0.75. (PDF) [file pone.0221880.s006.pdf]
